# Solubilization of Tea Seed Oil in a Food-Grade Water-Dilutable Microemulsion

**DOI:** 10.1371/journal.pone.0127291

**Published:** 2015-05-21

**Authors:** Lingli Deng, Fei Que, Hewen Wei, Guangwei Xu, Xiaowei Dong, Hui Zhang

**Affiliations:** 1 College of Biosystems Engineering and Food Science, Fuli Institute of Food Science, Zhejiang Key Laboratory for Agro-Food Processing, Zhejiang R&D Center for Food Technology and Equipment, Zhejiang University, Hangzhou 310058, China; 2 Department of Applied Engineering, Zhejiang Economic and Trade Polytechnic, Hangzhou 310018, China; 3 Jinhua Supervising and Testing Institute of Quality and Technology, Jinhua 321000, China; King's College London, UNITED KINGDOM

## Abstract

Food-grade microemulsions containing oleic acid, ethanol, Tween 20, and water were formulated as a carrier system for tea seed oil (*Camellia oleifera* Abel.). The effect of ethanol on the phase behavior of the microemulsion system was clearly reflected in pseudo-ternary diagrams. The solubilization capacity and solubilization efficiency of tea seed oil dispersions were measured along the dilution line at a 70/30 surfactant/oil mass ratio with Tween 20 as the surfactant and oleic acid and ethanol (1:3, w/w) as the oil phase. The dispersed phase of the microemulsion (1.5% weight ratio of tea seed oil to the total amount of oil, surfactant, and tea seed oil) could be fully diluted with water without phase separation. Differential scanning calorimetry and viscosity measurements indicated that both the carrier and solubilized systems underwent a similar microstructure transition upon dilution. The dispersion phases gradually inverted from the water-in-oil phase (< 35% water) to the bicontinuous phase (40–45% water) and finally to the oil-in-water phase (> 45% water) along the dilution line.

## Introduction

Microemulsions are defined as mixtures of water, oil, surfactant, and optional cosurfactant that are thermodynamically stable, transparent, isotropic, and dispersed [1]. Due to their nanoscale size (5–100 nm), microemulsions have found considerable applications in a wide variety of industries, including the pharmaceutical, cosmetics, and oil recovery industries [2–4]. Currently, food-grade microemulsions have attracted much attention due to their potential applications in the food industry [5, 6].

As a good solvent for lipophilic functional components, triglycerides have been widely used in carrier systems for drugs and bioactive nutrients [5, 7]. A fundamental study conducted by Parris et al. [8] showed that triglycerides containing unsaturated or short-chain fatty acids exhibited better dispersion properties in microemulsions than did triglycerides with saturated or long-chain fatty acids. However, it has been observed that the solubilization capacity of microemulsions increases with the chain length of surfactant molecules [9]. In recent years, several reports focusing on triglyceride microemulsions have been published. Winsor Type III and IV microemulsions have been successfully constructed using linear alkyl polypropoxylated sulfate (LAPS) surfactants, lipophilic linkers, hydrophilic linkers, vegetable oil, and water [10]. The results obtained by Djekic et al. [11] suggested that dilutable olive oil microemulsions could only be formed at a surfactant-to-cosurfactant mass ratio of 1:9, whereas a medium-chain triglyceride was easier to disperse as a microemulsion. In addition, a recent publication successfully developed a dilutable microemulsion composed of soybean oil, ionic liquid, TX-100, and n-butanol [12]. Although some dilutable triglyceride microemulsions have been prepared as described above, their applications in food are still limited to non-food grade surfactants or co-surfactants. Indeed, the development of a long-chain triglyceride for the formation of a microemulsion that can be diluted in water without phase separation is challenging using only food-grade surfactants.

Tea seed oil from *Camellia oleifera* Abel is commonly used as a cooking oil in China, Japan and India. Besides, it has been traditionally used as a medicine in history to treat stomachache and burning injuries in South Asian countries [13]. Its triglyceride profile consists of 56% oleic acid (C18:1), 22% linoleic acid (C18:2), and 0.3% linolenic acid (C18:3) and is therefore similar to that of olive oil [14]. Tea seed oil provides a number of health benefits because it can act as a prophylactic agent to prevent free radical related diseases [15, 16]. For example, Zhang and Zhou [17] reported the antioxidant ability of tea seed oil to reduce liver reactive oxygen species in rats. Despite excellent functional properties, to date, the application of tea seed oil is still restricted. However, microemulsification is a creative way to expand its application range in the food industry, as well as to improve its added value.

The objective of this study was to construct a food-grade carrier microemulsion for the solubilization of tea seed oil. Because vehicles for food supplements must be diluted with an aqueous phase, our goal was to make our tea sea oil microemulsion able to undergo full dilution. The carrier microemulsion system was composed of Tween 20 as the surfactant phase, oleic acid and optional ethanol as the oil phase, and water, and the system was studied using pseudo-ternary diagrams. Differential scanning calorimetry (DSC) and viscosity measurements were performed to study the microstructural transitions of the carrier and solubilized systems during water dilution.

## Materials and Methods

### Materials

Tween 20, oleic acid, and ethanol were purchased from Sinopharm Chemical Reagent Co., Ltd., China. The transparent light yellow tea seed oil (Extra Virgin, Brand Jinlongyu, Shanghai, China) was purchased from a local Walmart (geographical coordinates: 30°18′N, 120°06′E). Its triglyceride composition was 81.4% oleic acid, 7.9% linoleic acid, 7.7% palmitic acid, 1.7% stearic acid, and 0.23% linolenic acid based on the preliminary gas chromatography analysis. Double-distilled water was used in this study. All reagents were used without further purification.

### Phase diagrams

Pseudo-ternary phase diagrams were constructed in order to examine the behavior of the multi-component microemulsion system. Surfactant-oil phase mixtures with a predetermined weight ratio of oil phase (ethanol plus oleic acid) to Tween 20 were prepared in glass test tubes, which were sealed with caps and stored at 25°C. Water was titrated into the oil-surfactant mixtures with vigorous shaking using a stirrer plate. The prepared samples were held at 25°C for equilibration for at least 24 h prior to measurements. The samples, which remained homogeneous and visually transparent after vigorous vortexing, were regarded as microemulsions. The accuracy of the phase boundaries was within 4 wt%.

The total monophasic area (A_T_), as well as the maximal amount of aqueous phase (W_M_) of each phase diagram, was calculated according to the method described in our previous study [18]. Briefly, the total monophasic area (A_T_) was calculated by the percentage of the integral monophasic area in the total area of phase diagram. W_M_ is the maximum amount of water content, where one-phase region transforms into multi-phase regions.

### Solubilization measurements

The solubilization measurements were conducted based on the method of Amar et al. [19], with some modifications. Different amounts of tea seed oil were titrated into a reverse micelle system composed of oleic acid, ethanol, and Tween 20, followed by water dilution to a defined aqueous content. The samples were ultrasonicated at 40 kHz with an intensity of 250 W in an ultrasonicator (KQ-250DE, Kunshan Ultrasonic Instrument Co., Ltd, Shanghai, China) for 5 min at 25°C to accelerate the solubilization and then stored at 25°C. Samples that remained visually transparent for at least 7 days were regarded as microemulsions.

The effect of water dilution on the solubilization was represented by two parameters that reflect the effect of structural change in microemulsions upon solubilization, as suggested by Amar et al. [19]. The first parameter is the “solubilization capacity”, which represents the maximum solubilization amount of tea seed oil (in ppm). This relationship is important because it demonstrates how much tea seed oil can be solubilized per preparation. The second parameter is the “solubilization efficiency”, which represents the maximum amount of tea seed oil solubilized in the microemulsion normalized to the amount of oil and surfactant (in this case, oleic acid, ethanol, and Tween 20) at each dilution point (in ppm). Evaluation of the solubilization efficiency is also important because microemulsions are usually transported and stored in a concentrated form and later diluted with water.

### Viscosity measurements

Viscosity measurements were conducted on a viscometer (NDJ-8S, Heng Yuan Equipment Co., Ltd., Hangzhou, China) at 25°C. A steady shear was applied at 30 rpm. All measurements were conducted in triplicate.

### Differential scanning calorimetry (DSC) measurements

DSC analysis was conducted on a Q200 differential scanning calorimeter (TA Instruments, New Castle, Delaware, U.S.A.) using our previously reported procedures [20]. Briefly, the microemulsion samples were carefully weighed into standard 40 μL aluminum pans and sealed immediately. Pans were quickly cooled from ambient temperature to -90°C; after equilibrating at -90°C for 20 min, the samples were heated from -90°C to 40°C at a rate of 5°C/min. An empty aluminum pan was used as a reference. The thermal behaviors reflecting different water states were analyzed, and the contents of different states of water were calculated by the method established by Senatra et al. [21].

## Results and Discussion

### Phase diagrams

**Fig 1:**
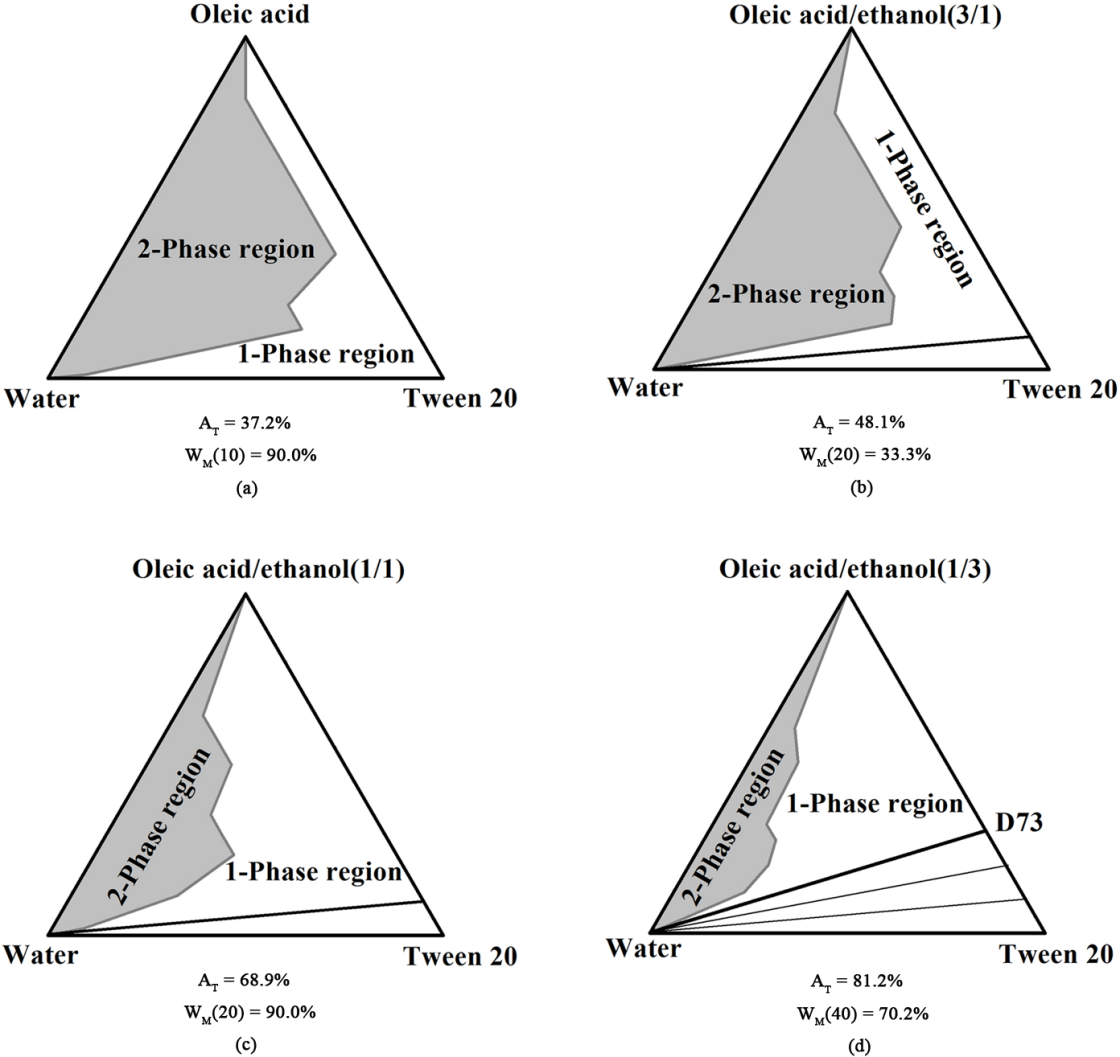
Pseudo-ternary phase diagrams for microemulsions. Pseudo-ternary phase diagrams for microemulsions of oleic acid, ethanol, Tween 20, and water. The oil phase used were (a) oleic acid; (b) oleic acid/ethanol = 3:1; (c) oleic acid/ethanol = 1:1; (d) oleic acid/ethanol = 1:3.

Phase diagrams of the multi-component microemulsions containing different weight ratios of oleic acid/ethanol were studied. The microemulsion region area was strongly affected by the addition of ethanol, as shown in Fig 1. In the phase diagram comprising oleic acid, Tween 20, and water (Fig 1A), a small one-phase region [A_T_ = 37.2%; W_M_(10) = 90.0%] was formed without any effective dilution line. A dramatic increase in the one-phase area was observed with the addition of ethanol. When a 1:3 weight ratio of ethanol to oleic acid was used (Fig 1B), the one-phase region increased [A_T_ = 48.1%], and one effective dilution line appeared. When the weight ratio of oleic acid to ethanol was 1:1, the area of the one-phase region increased further [A_T_ = 68.9%; W_M_(20) = 90.0%] (Fig 1C). When the ratio of oleic acid/ethanol was 1:3, the area of the two-phase region decreased drastically [A_T_ = 81.2%; W_M_(40) = 70.2%], and the diluting channels were significantly broadened (Fig 1D).

It has been reported that ethanol can modify the polarity of molecules and improve the interface flexibility of microemulsions [22]. In addition, it has been reported that the addition of ethanol improves the solution properties of surfactants [23]. One possible interpretation for the increase in the one-phase area that we observed is that increased incorporation of ethanol into the oil-water interface led to an increase in the mobility of the interfacial layer. Collectively, these observations suggest that the increased penetration of the surfactant film and the decreased polarity of water favor the formation of microemulsions [24].

### Solubilization measurements

**Fig 2:**
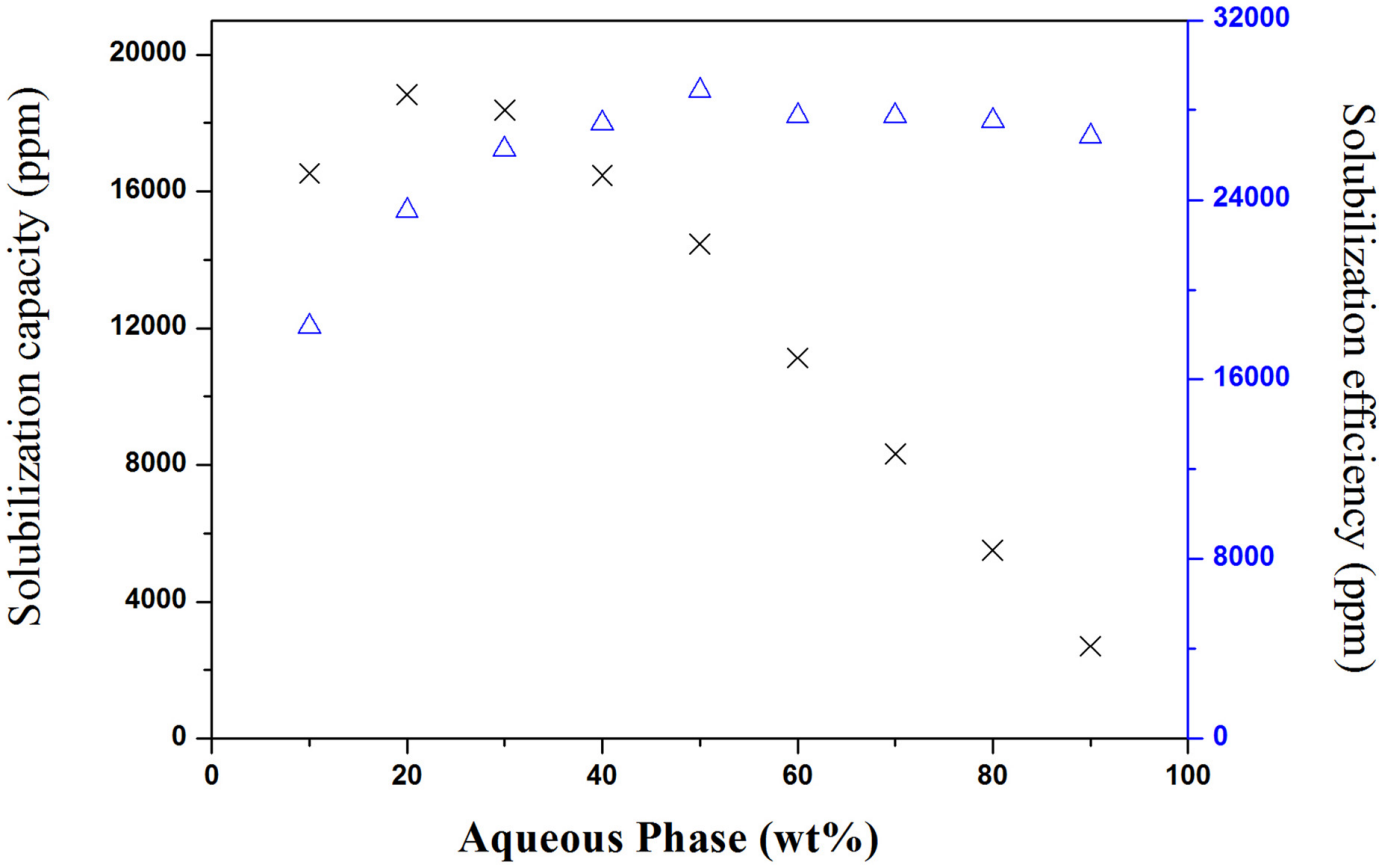
Solubilization capacity and solubilization efficiency of tea seed oil. The solubilization capacity of tea seed oil (amount of tea seed oil per preparation) (×), and solubilization efficiency (amount of tea seed oil normalized to the amount of oil, ethanol, and surfactant) (∆) were plotted against the aqueous phase content, along the dilution line D73 at 25°C.

The solubilization of tea seed oil in a carrier microemulsion system along the dilution line D73 (Fig 1D) was investigated. The effect of water dilution on the solubilization capacity and solubilization efficiency was shown in Fig 2. It should be noted that the solubilization capacity of tea seed oil in a reverse micelle is 39,850 ppm, which is more than two-fold greater than the solubilization capacity of the microemulsion at 10% water content (16,530 ppm). In a reverse micelle, tea seed oil could accumulate in the core of the micelle and on the interface. Based on the significant decrease in solubilization capacity and solubilization efficiency, we suspect that once water was added, the core of the micelle was occupied by water and the hydrophobic tea seed oil could only accumulate at the interface.

At 20% aqueous phase, the solubilization capacity increased slightly to 18,840 ppm, and the solubilization efficiency increased from 18,363 ppm to 23,520 ppm, despite the dilution effect. With addition of water, a slightly enhanced solubilization capacity was observed, likely due to the consumption of the tea seed oil by the interface. It could be inferred that once the water-in-oil (w/o) microemulsion droplets grew, more tea seed oil could be accommodated on the interface, resulting in an increase in solubilization capacity and efficiency. At this stage, the interfacial effect was greater than the dilution effect.

Between water contents of 30 and 50%, the solubilization capability decreased from 18,375 to 14,470 ppm and the solubilization efficiency increased from 26,250 to 28,850 ppm. It is assumed that most of water was bound to the ethanol and the hydroxyl groups of the surfactant located at the interface. When water was entrapped in the core, the micelle swelled, the interfacial area increased, so more surfactant and cosurfactant migrated to the interface. Once the microemulsion started to invert from a w/o structure to a bicontinuous phase, tea seed oil was more easily accommodated at the interface, thus, its solubilization efficiency increased significantly (up to a maximum value of 28,000 ppm). The opposite trend of the solubilization capacity and efficiency suggested that the dilution effect was greater than the interfacial effect.

With the increase in water content from 60 to 90%, a significant decrease in solubilization capacity (reduction to 2700 ppm at 90% water content) was observed, but the solubilization efficiency did not change appreciably. The oil-in-water (o/w) microemulsion was formed by water dilution, and tea seed oil was not as well accommodated at the oil-water interface as it was previously, leading to a slight decrease in the solubilization efficiency compared to that of the microemulsion at 50% water content. At this stage, the interfacial area remained nearly unchanged, but a dilution effect resulted in a significant decrease in solubilization capacity.

Spernath et al. [25] conducted lycopene solubilization in a food-grade microemulsion and found that solubilization capacity and efficiency were microstructure-dependent, but their results showed the highest solubilization efficiency at o/w region, which is approximately 8000 ppm. Garti et al. [26] solubilized lutein in U-type microemulsions and found that transitions from w/o structure to bicontinuous structure led to the highest solubilization efficiency of lutein (approximately 20,000 ppm), which then dropped significantly once the o/w structure was formed. It can be concluded that the solubilization capacity and the solubilization efficiency was greatly affected by the interfacial microstructure of the microemulsions. A water-dilutable tea seed oil microemulsion with a constant solubilization efficiency of 1.5% was chosen to further study the microstructure transitions.

### Differential scanning calorimeter measurements

**Fig 3:**
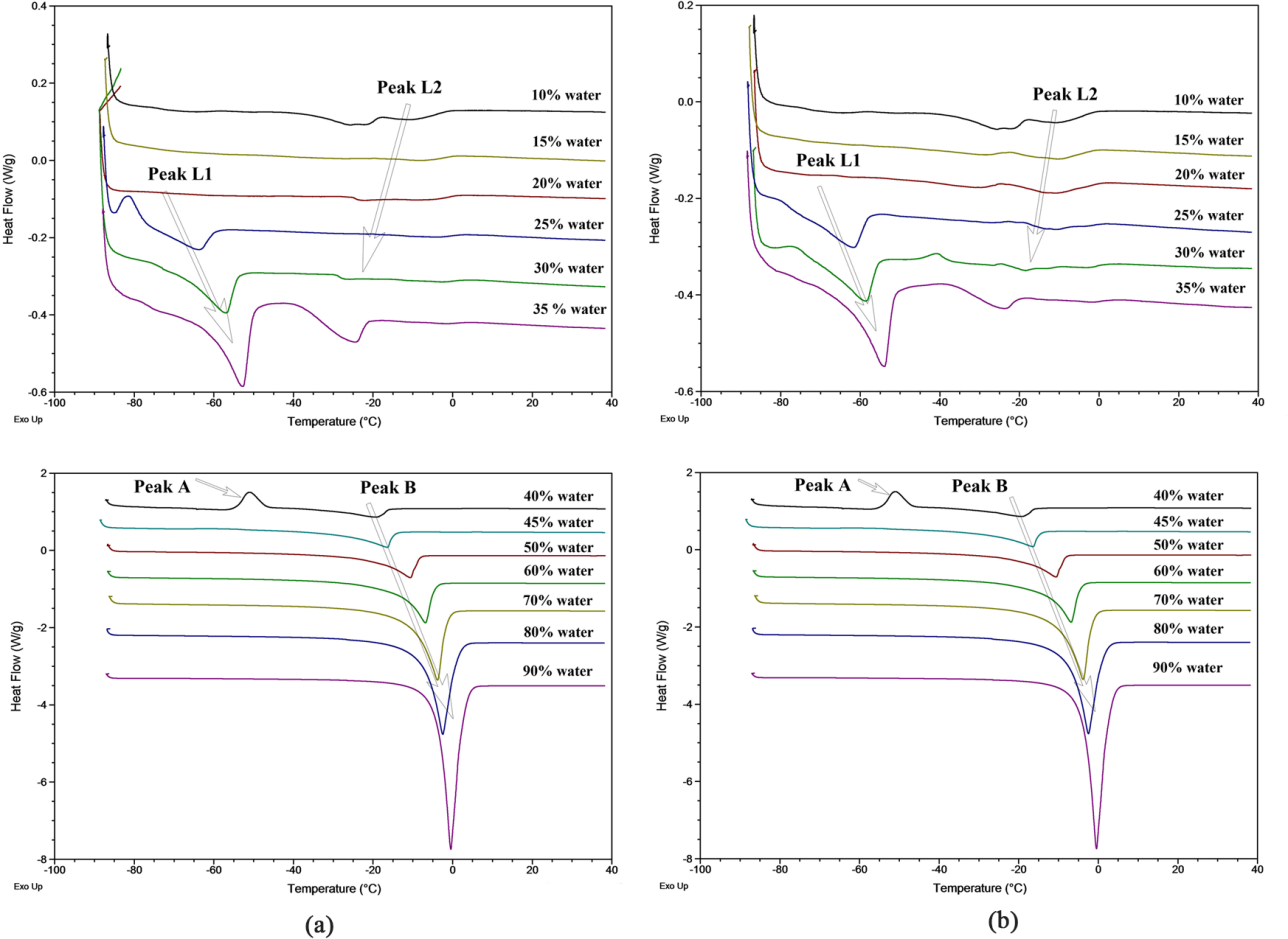
DSC curves of carrier and solubilized systems. DSC curves of microemulsions along dilution line D73: (a) the carrier system; (b) the solubilized system (1.5% weight ratio of tea seed oil to the total amount of oil and surfactant).

DSC has been widely used for studying binary and multicomponent systems containing surfactant [27–29]. The technique was used in this work to study the microstructure transition of the carrier system along dilution line D73 and a tea seed oil microemulsion with a constant solubilization efficiency of 1.5%. According to the theory of Senatra et al. [21], three types of water could be differentiated based on the water melting point: (i) free water, which melts at approximately 0°C; (ii) interfacial water, defined as water confined within the interface of the dispersed system (melts at approximately -10°C); and (iii) bound water, which is associated with hydrophilic groups (melts at < -10°C). Therefore, water in this system could be divided into three stages based on the thermal behaviors of the carrier (Fig 3A and Table 1) and solubilized systems (Fig 3B and Table 2).

**Table 1:** Thermal behavior of the carrier system along the dilution line D73 at different water contents measured by DSC.

Water content/mass%	Peak L1^a^		Peak L2^a^		Peak A^b^		Peak B^c^	
	T/°C	∆*H*_F_ / J g^-1^	T/°C	∆*H*_F_ / J g^-1^	T/°C	∆*H*_F_ / J g^-1^	T/°C	∆*H*_F_ / J g^-1^
10	-	-	-13.80	-3.85	-	-	-	-
15	-	-	-8.68	-3.91	-	-	-	-
20	-	-	-12.78	-0.50	-	-	-	-
25	-63.83	-5.25	-9.44	-2.59	-	-	-	-
30	-56.94	-9.71	-26.59	-1.93	-	-	-	-
35	-52.28	-15.51	-26.46	-8.25	-	-	-	-
40	-	-	-	-	-48.95	26.89	-18.49	-29.99
45	-	-	-	-	-	-	-16.65	-42.02
50	-	-	-	-	-	-	-11.92	-55.97
60	-	-	-	-	-	-	-7.43	-86.27
70	-	-	-	-	-	-	-4.28	-132.90
80	-	-	-	-	-	-	-2.27	-188.10
90	-	-	-	-	-	-	-0.53	-243.10

^a^Peaks were interpreted as the melting of the surfactant mixture.

^b^Peak A was interpreted as the recrystallization of the bound water.

^c^Peak B was interpreted as the melting point of water.

**Table 2:** Thermal behavior of the solubilized system along dilution line D73 at different water contents measured by DSC.

Water content/mass%	Peak L1^a^		Peak L2^a^		Peak A^b^		Peak B^c^	
	T/°C	∆*H*_F_ / J g^-1^	T/°C	∆*H*_F_ / J g^-1^	T/°C	∆*H*_F_ / J g^-1^	T/°C	∆*H*_F_ / J g^-1^
10	-	-	-8.47	-1.39	-	-	-	-
15	-	-	-9.88	-2.39	-	-	-	-
20	-	-	-10.80	-3.22	-	-	-	-
25	-61.67	-7.58	-11.00	-1.93	-	-	-	-
30	-68.97	-8.69	-18.53	-2.24	-	-	-	-
35	-53.96	-16.46	-25.23	-4.09	-	-	-	-
40	-	-	-	-	-51.09	24.18	-19.81	-27.33
45	-	-	-	-	-	-	-16.46	-41.62
50	-	-	-	-	-	-	-10.72	-56.18
60	-	-	-	-	-	-	-6.87	-89.10
70	-	-	-	-	-	-	-3.84	-136.30
80	-	-	-	-	-	-	-2.47	-180.10
90	-	-	-	-	-	-	-0.45	-242.70

^a^Peaks were interpreted as the melting of the surfactant mixture.

^b^Peak A was interpreted as the recrystallization of the bound water.

^c^Peak B was interpreted as the melting point of water.

In the first stage (0–35% water), two endothermic peaks were observed in both systems, indicating a similar thermal behavior. Peak L2 varied from -26.59 to -8.68°C in the carrier system and from -25.23 to -8.47°C in the solubilized system. Peak L1 varied from -63.83 to -52.28°C and from -61.67 to -53.96°C in the carrier and solubilized system, respectively. Clearly, these two endothermic peaks do not reflect ice melting to water, peak L2 may be related to the thermal behavior of the surfactant-oil phase [23]. However, because the peak temperature and heat fusion of peak L1 increased with the water content, water may have been contained in the regular structure of the microemulsion. It should be noted that at this stage, no thermal transitions of water were detected, which suggests that the water activity was below the identification capability of DSC. This type of water is termed “non-freezable water” or “non-detectable” water [21].

In the second stage (35–40% water), peak L1 and peak L2 disappeared, but an exothermic event and an endothermic event occurred. As reported by Spernath et al. [30], this exothermic peak (peak A) was identified as a water-related event. The entropy change at peak A (24.18 J g^-1^) was identical to that at peak B (-27.33 J g^-1^) in the solubilized system, which suggests that the endothermic event (peak B) should be attributed to a structural change in water. The carrier system at 40% water content showed the similar thermal behavior. With the increase in water content, the mobility and activity of the water increased, and the water could be identified as bound water.

In the third stage (45–90% water), with the disappearance of peak A, peak B became the dominant peak, and its ∆*H*_F_ increased with the water content. In the microemulsion with high water content, the detection of other thermal events became impossible due to shielding by the mass of water. This finding suggests that the water was slowly transformed from bound water into interfacial water, or alternatively, the free water interacted with micelles.

**Fig 4:**
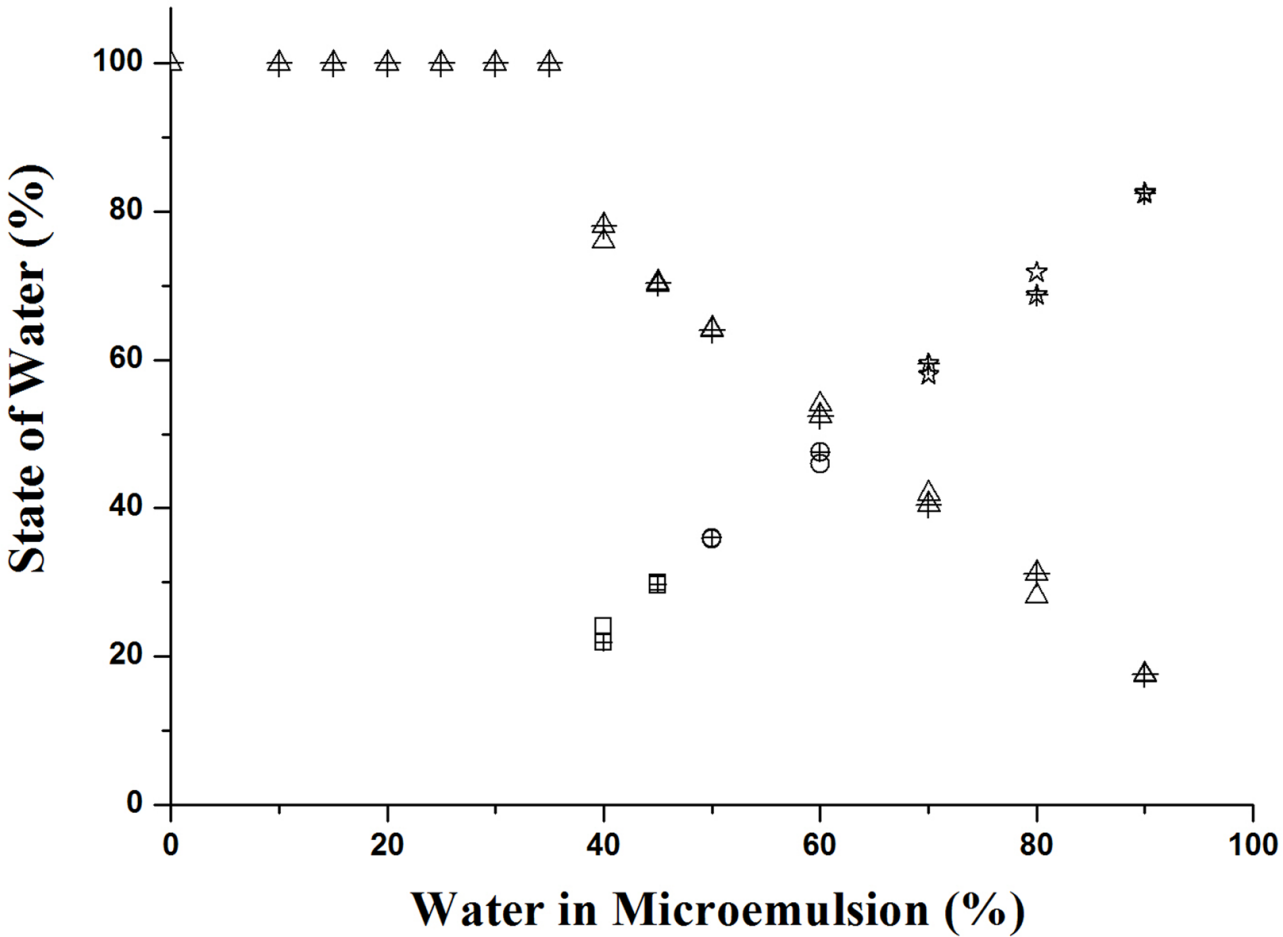
The contents of water at different states in carrier and solubilized systems. Variation in the content of non-freezable (∆), bound (□), interfacial (○), and free water (☆) as a function of water content along dilution line D73 for the (a) carrier (hollow symbol), and (b) solubilized systems (hollow plus cross symbol). Some data points of the carrier and solubilized systems are totally overlapped with each other.

The equations established by Yaghmur et al. [31] were applied to identify various states of water in these carrier and solubilized systems. From Fig 4, it can be inferred that water was confined to the core of the reverse micelle and bound to the surfactant head groups in systems containing no more than 35% water. In systems containing 40–45% water, the transformation from non-freezable water to bound water may have been due to the saturation of the surfactant head group by water molecules. Thus, excess water molecules with relative high activity were bound to the amphiphilic film by hydrophobic effect and dipolar forces. A further increase in water content in the microemulsion systems (50 to 60% water) caused a further decrease in the concentration of non-freezable water. The activity of the non-freezable water appeared to be higher than the activity of bound water, thus, the former is termed “interfacial water”. At higher water contents (over 70%), water in the microemulsions appeared to be free water, as the ∆*H*_F_ value approached that of pure water.

The change in the states of water (Fig 4) showed that solubilization of tea seed oil did not alter the microstructure transitions of the carrier microemulsion system. In the first stage, at water contents of up to 35%, w/o microemulsions were formed and all water molecules were confined to the water core of the reverse micelles. At the water contents of 40–45%, the microstructure transformed into a bicontinuous phase due to the increase in the mobility of water, and finally, the microemulsions adopted an o/w structure in the third stage (above 45% water).

### Viscosity measurements

**Fig 5:**
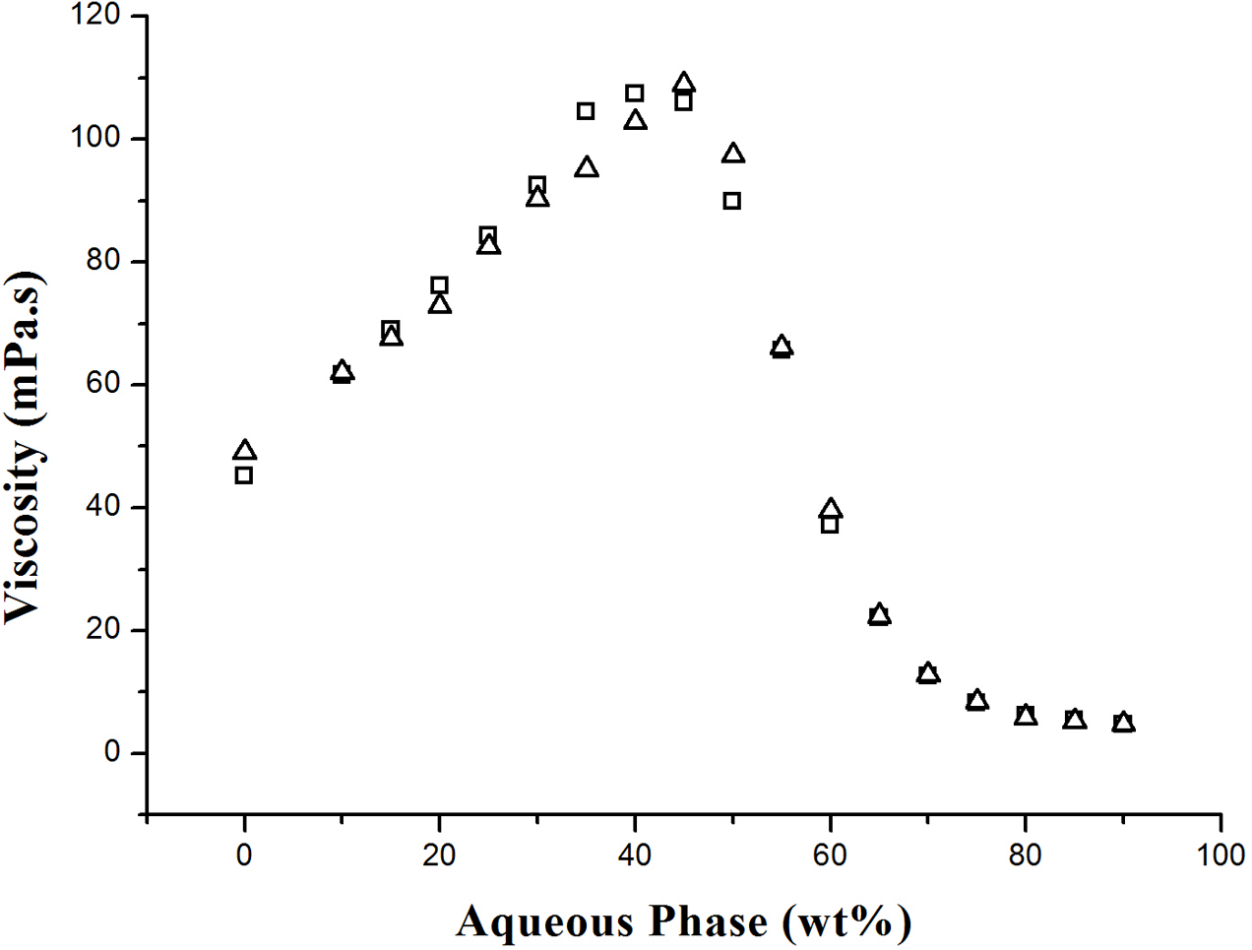
The viscosity of carrier and solubilized systems. The viscosity of microemulsions along dilution line D73 for the carrier (∆) and solubilized (□) systems.

It has been well documented that microemulsions exhibit Newtonian behavior and their viscosity is structure-dependent [19, 32, 33]. A plot of viscosity profiles as a function of the aqueous phase weight fractions along the dilution line D73 is shown in Fig 5. The viscosity curves for both the carrier and solubilized systems are similarly bell-shaped.

With a small fraction of water, globular micelles confining water molecules in the continuous surfactant-oil mixture demonstrates that the interactions between the globules were weak; these properties are reflected in the low-viscosity values [34]. When the water content increased to 35%, the viscosity increased from 49.1 to 95.1 mPa s in the carrier system and from 45.2 to 104.5 mPa s in the solubilized system. The viscosity increase of the w/o microemulsions was due to an increase in the dispersed droplet size and the enhanced attractive interactions between the droplets [22, 35]. With the increase in the aqueous phase content, bicontinuous structures were progressively formed. In this region, the interconnected water and oil “channels” progressively enhanced structural interactions up to a maximum viscosity at 40% aqueous phase in the solubilized system and 45% in the carrier system. However, with further water dilution, the bicontinuous structure gradually collapsed, and a transition to an o/w microemulsion occurred, which was reflected by a sharp decrease in viscosity. In this aqueous-rich solution, the viscosity gradually decreased due to a dilution effect of the o/w microemulsion droplets, and finally, the viscosity of a highly diluted o/w microemulsion reached a constant value of approximately 5 mPa s.

The general viscosity patterns of both the carrier and solubilized microemulsions appeared to be similar, except for slight differences in the peaks of the curves. The peak of the carrier system occurred at 45%, whereas that of the solubilized system occurred at 40%. At water contents of 35–40%, the solubilized system was slightly more viscous than the carrier system. It appears that the tea seed oil “pulled” the surfactant toward the interface and enhanced the interactions between droplets, produced a higher degree of tail entanglement, and gave rise to a higher viscosity [36]. This effect may have also been reflected by the earlier structural inversion from the bicontinuous structure to the o/w microemulsion by triglyceride molecules of tea seed oil. The slightly shift of viscosity peak after solubilizing guest molecules was also observed in many other studies. Amar et al. [19] found that the maximum viscosity of the microemulsion containing free lutein was impeded to 50% aqueous phase in comparison to that of an empty microemulsion, which occurred at 45%. Garti et al. [26] reported a viscosity shift from 45% to 48% water content after solubilizing phytosterols in U-type microemulsions consist of Tween 60, R (+)-limonene, ethanol, propylene glycol, and water. These results suggested that solubilized molecules could alter the interfacial curvature, and retard or accelerate the formation of o/w structures.

## Conclusions

We successfully constructed a food-grade water-dilutable carrier microemulsion system for the solubilization of tea seed oil. The carrier system was composed of oleic acid, ethanol, Tween 20, and water and proved to be a suitable vehicle. The solubilization capacity was greatly affected by the dilution of water, but the solubilization did not change the microstructural transitions of the microemulsion system during dilution, as indicated by DSC and viscosity measurements.

Our work suggests the possibility of applying tea seed oil in the production of beverages without phase separation. The temperature stability and salt tolerance of such systems in beverage models will be studied in the future.
